# Significant Artifact Reduction at 1.5T and 3T MRI by the Use of a Cochlear Implant with Removable Magnet: An Experimental Human Cadaver Study

**DOI:** 10.1371/journal.pone.0132483

**Published:** 2015-07-22

**Authors:** Franca Wagner, Wilhelm Wimmer, Lars Leidolt, Mattheus Vischer, Stefan Weder, Roland Wiest, Georgios Mantokoudis, Marco D. Caversaccio

**Affiliations:** 1 University Department of Diagnostic and Interventional Neuroradiology, Inselspital Bern, Bern, Switzerland; 2 University Department of Otorhinolaryngology, Head & Neck Surgery, Inselspital Bern, Bern, Switzerland; 3 ARTORG Center for Biomedical Engineering Research, University of Bern, Bern, Switzerland; University of North Carolina at Chapel Hill, UNITED STATES

## Abstract

**Objective:**

Cochlear implants (CIs) are standard treatment for postlingually deafened individuals and prelingually deafened children. This human cadaver study evaluated diagnostic usefulness, image quality and artifacts in 1.5T and 3T magnetic resonance (MR) brain scans after CI with a removable magnet.

**Methods:**

Three criteria (diagnostic usefulness, image quality, artifacts) were assessed at 1.5T and 3T in five cadaver heads with CI. The brain magnetic resonance scans were performed with and without the magnet in situ. The criteria were analyzed by two blinded neuroradiologists, with focus on image distortion and limitation of the diagnostic value of the acquired MR images.

**Results:**

MR images with the magnet in situ were all compromised by artifacts caused by the CI. After removal of the magnet, MR scans showed an unequivocal artifact reduction with significant improvement of the image quality and diagnostic usefulness, both at 1.5T and 3T. Visibility of the brain stem, cerebellopontine angle, and parieto-occipital lobe ipsilateral to the CI increased significantly after magnet removal.

**Conclusions:**

The results indicate the possible advantages for 1.5T and 3T MR scanning of the brain in CI carriers with removable magnets. Our findings support use of CIs with removable magnets, especially in patients with chronic intracranial pathologies.

## Introduction

Cochlear implants (CIs) have become a highly standardized and safe treatment for hearing restoration in prelingually deafened children and postlingually deafened children or adults. The indications for CI implantations are constantly expanding. Patients receiving a CI might have three major disadvantages regarding magnetic resonance (MR) imaging: 1) the magnetic receiver coil under the skin might cause pain during the acquisition of the MR images, 2) dislocation of the implant is possible, and 3) the internal magnet creates large artifacts that limit the diagnostic validity. MR imaging (MRI) is often required for brain, head and neck examinations for a prolonged period following CI implantation. One study predicted that the lifetime prevalence of neurological disorders requiring brain MR imaging in patients with CI implants is 6.25 percent [1], so this issue affects a very large number of patients. About 10% of cochlear carriers in developed countries can be expected to undergo MRI within their lifetimes [2].

The indication for CI expanded from single-sided implantation to bilateral implants, and from elderly patients with single-sided deafness to those with residual hearing. For many years patients with CI could not undergo MRI examinations. However in the last 15 years, several studies by different research teams and manufacturers have shown no major incidents in MRI examinations using field strengths up to 1.5T. This was first evaluated with in vitro studies [3–7], then in cadaveric studies or in studies involving only a few patients [8–11], followed by studies involving larger numbers of patients [12–15]. Finally, several authors concluded that MRI scans up to 1.5T are safe in patients carrying CIs with non-removable magnets if strict guidelines are followed [16–18]. With the continuing trend towards higher field strength MRI, the compatibility of CIs with 3T MRI is important.

MR imaging in CI patients also raises concerns regarding MRI-induced artifacts caused by the implant [14,16,19,20]. A CI includes a removable external speech processor and transmitter coupled transcutaneously to a surgically implanted component. A small internal CI magnet is used to magnetically hold the external part (audio processor coil) safely over the implant, which guarantees sufficient transmission quality between the external transmitter and the internal receiver [21]. Artifacts are induced by both the metallic parts of the CI and the magnet inside the implant. Therefore many physicians consider this a contraindication to MRI, especially in units operating at high-field strength [14,16,19,20]. Thus, the artifacts caused by CIs in brain MR imaging remain a concern.

**Fig 1:**
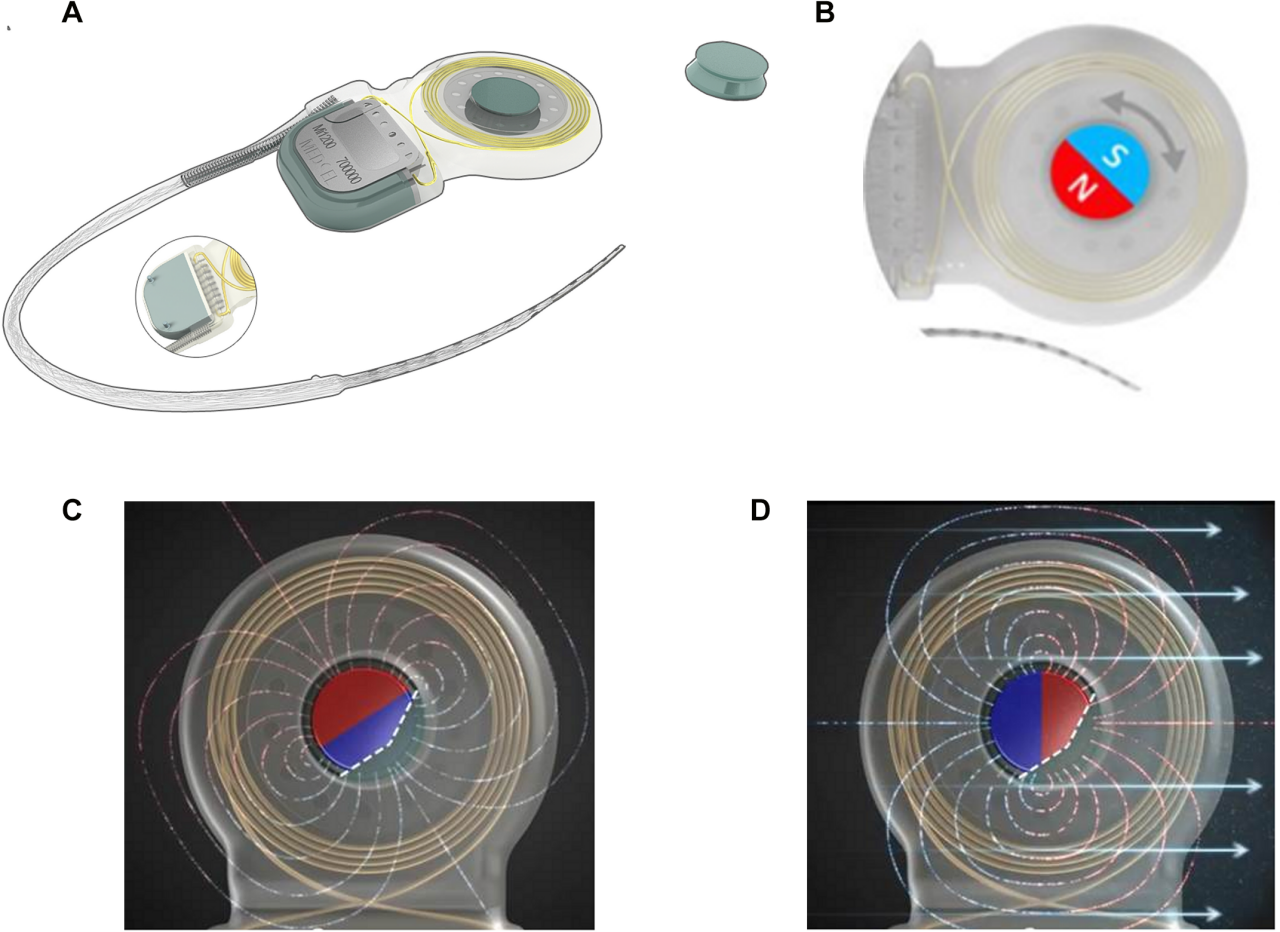
The MED-EL Mi1200 SYNCHRONY Cochlear Implant (MED-EL, Innsbruck, Austria). The MED-EL Mi1200 SYNCHRONY Cochlear Implant (MED-EL, Innsbruck, Austria) is compatible with MRI at a magnetic-field strength of up to 3T and with the possibility of removing the internal magnet (1a). The magnet is freely rotating (1b). In Fig 1C the magnet is in a random position, when no external magnetic field is applied and in contrast in Fig 1D the magnet aligned parallel to the external magnetic field.

One solution to reduce artifacts and to increase patient safety was proposed by the company MED-EL (MED-EL Corporation, Innsbruck, Austria), which is developing implants with removable magnets. MED-EL implants have long been CE (Conformité Européenne)-approved for MRI at field-strengths of 0.2T, 1.0T and 1.5T [5,6,12,14,20]. For countries accepting the CE mark and FDA approval, the Mi1200 SYNCHRONY CI (Fig 1) is CE-approved and labeled as MR conditional for MRI at magnetic field strengths of 0.2T, 1.0T, 1.5T and 3T, and labeled as MR conditional for examinations at 1.5T and 3T magnetic field strengths. This is due to a design that allows the implant magnet to freely rotate and self-align within its silastic housing, reducing implant torque and the risk of demagnetization during MR scans. The implant also offers the possibility of removing the magnet in case of large imaging artifact (Fig 1).

Other cochlear implant manufacturers also offer devices that enable scanning procedures at 3T. Cochlear (Sydney, Australia) offers a 3T-approved device with the possibility of magnet removal to decrease MRI-related artifacts. A similar product is offered by Advanced Bionics (Stäfa, Switzerland), which has approval for up to 1.5T. Neurelec’s device (Oticon Medical, Askim, Sweden) is approved for up to 1.5T in a ceramic housing, even with a non-removable magnet.

The primary aim of this human cadaveric study was to determine to what extent MR brain artifacts are reduced after explantation of the removable magnet of the MED-EL Mi1200 SYNCHRONY cochlear implant at 1.5T and at 3T with different MR sequences. Secondary objectives were to determine to what extent the magnet in situ affects the diagnostic usefulness and image quality at 1.5T and at 3T.

## Materials and Methods

This experimental study was designed and conducted according to the guidelines of the Cantonal Ethics Committee and the Cantonal Animal Experimentation Commission of Bern. Our institutional review board–the Head Office Education and Research–approved this study (# 2604).

The Institute of Anatomy, University of Bern, Switzerland (http://www.ana.unibe.ch) received written informed consent from all donors. This is a legal requirement and serves as liable document to justify any donation and to further guarantee that the act of donation meets all legal requirements. Furthermore, the written consent allowed us to use human body parts for research with human material according to Swiss jurisdiction. The specimens used in this study have not been used or described in previous publications.

### Anatomical specimens and surgical procedure

In order to accurately simulate an MR brain examination for evaluating intracranial pathology in a human, the CI was implanted in five Thiel-fixed [22] cadaver heads. The Thiel conservation technique is considered to realistically replicate the color, mobility and flexibility of tissues in intraoperative situations and to offer excellent conditions for the training of surgical procedures in otology [22]. In contrast to the frequently used formaldehyde fixation for anatomic specimens, the Thiel solutions are adapted to individual organ systems. Structure and consistency of the tissues of the auditory canal, the tympanic cavity and the mastoid are comparable to vital conditions [22]. This enabled a surgical preparation under conditions close to the intravital situation.

The five cadaver heads were obtained from the Institute of Anatomy, University of Bern. First, in all five cadavers a native 1.5T and 3T baseline MR imaging was performed to verify sufficient conservation of the anatomical structures and to exclude any other lesions (e.g. tumors, large hemorrhage or advanced putrefaction) that might affect the study. To avoid accumulation of air bubbles in the heads, especially in parieto-occipital spaces and in the cerebellopontine angle, the heads were placed according to the normal supine position of a human head in MR for 48 hours before the MR examination. With this positioning the post-mortem intracranial air accumulated in the frontal lobes and the frontal subarachnoidal space.

All imaging studies were performed using 1.5T and 3T Siemens scanners (Magnetom Avanto, Magnetom Verio, respectively; Siemens Medical Solution, Erlangen, Germany) with 12-channel head coils. Our pre-implantation MRI protocol included T1-weighted (w) multiplanar (MPR) sequences, axial T1w and T2w sequences, a coronal T2w, an axial fat suppressed T1w, and an axial constructive interference in steady state (CISS), in accordance with our native temporal bone MR protocol. The corpse heads were supine positioned in the MR scanners according to the standard position in routine clinical practice.

### Imaging study protocol

First, all five corpse heads were scanned without the cochlear implant at 1.5T and 3T. Then the MR scanning was repeated with the entire implant and magnet in place. Finally the five corpse heads were MR scanned after explantation of the removable magnet. The MR study protocol at 1.5T and 3T for the non-implanted, implanted with magnet in situ, and implanted without magnet corpse heads was exactly the same. The chosen MR examination protocol corresponds exactly to our institutional protocol for the assessment of pathologies in the cerebellopontine angle and temporal bone without contrast application.

For the 1.5T MR scanner the following parameters were used: T1w multiplanar (MPR): repetition time (TR) 1830 ms, echo time (TE) 2.92 ms, slice thickness 1.0 mm and field of view (FoV) 256x256 mm^2^; acquisition time: 5 minutes (min) and 30 seconds (sec). Axial T1w: TR490 ms, TE 8.4 ms, slice thickness 5.0 mm and FoV 192x220 mm^2^; acquisition time: 2:13 min. Axial T2w: TR 4100 ms, TE 100 ms, slice thickness 5.0 mm and FoV 191x219 mm^2^; acquisition time: 1:51 min. Coronal T2w: TR 3500 ms, TE 98 ms, slice thickness 2.0 mm and FoV 180x180 mm^2^; acquisition time: 3:13 min. Axial T1w (fat suppression): TR 492 ms, TE 11 ms, slice thickness 2.0 mm and FoV 190x190 mm^2^; acquisition time: 1:34 min. CISS: TR 1200 ms, TE 264 ms, slice thickness 0.6 mm, FoV 200x200 mm^2^ and a flip angle 150°; acquisition time: 4:36 min.

Analogous to the 1.5T sequences, the 3T MRI sequences were implemented with the following parameters: T1w MPR: TR 2530 ms, TE 2.2 ms, slice thickness 1.0 mm and FoV 256x256 mm^2^; acquisition time: 3:58 min. Axial T1w: TR 250 ms, TE 3 ms, slice thickness 5.0 mm and FoV 192x220 mm^2^; acquisition time: 2:14 min. Axial T2w: TR 5270 ms, TE 86 ms, slice thickness 5.0 mm and FoV 192x220 mm^2^; acquisition time: 1:26 min. Coronal T2w: TR 4000 ms, TE 93 ms, slice thickness 3.0 mm and FoV 181x200 mm^2^; acquisition time: 3:14 min. Axial T1w (fat suppression): TR 319 ms, TE 8.6 ms, slice thickness 3.0 mm and FoV 198x220 mm^2^; acquisition time: 2:22 min. CISS: TR 1000 ms, TE 132 ms, slice thickness 0.5 mm, FoV 199x199 mm^2^ and a flip angle of 120°; acquisition time: 6:32 min.

### Surgical procedure for implant removal

A conventional transmastoid posterior tympanotomy approach was performed by five experienced otologists (GM, MV, SW and MDC). A bone bed for the CI body and a bony channel for the electrode cable were prepared behind the ear. The CI implant bodies were additionally fixed using resorbable sutures (Vicryl 2.0). The round window membrane was exposed by removal of the bony overhang and the electrode arrays were inserted through the round window into the cochlea. All electrode arrays were fully inserted without complications. Finally, a double-layered wound closure was performed. Three of the five corpse heads were implanted on the left side and the other two were implanted on the right. After the MR scans with the magnet in situ the magnet was explanted using a dedicated magnet removal tool via a small skin incision of 180 degrees around the receiver coil. One of the otologists did the incision for magnet exchange behind the ear beside the implant in all cases. After removal of the magnet a non-magnetic spacer (titanium shell without a magnet inside) was inserted as a placeholder with a dedicated insertion tool. The configuration of the implant with the non-magnetic spacer is hereafter referred to as “implant without magnet”.

### Imaging analysis

Evaluation of the images was performed by two experienced neuroradiologists (FW and LL), who reviewed the MR images independently. The acquired MR images were rated on certified reporting stations (DIN V 6868–57 and QA guideline). Three criteria–diagnostic usefulness, artifacts, and image quality–were defined and assessed on scales of 1 to 4 (Table 1). The following brain structures were evaluated and graded on the ipsilateral CI side with regard to the three defined criteria: temporal, parietal and occipital lobe; cerebellum (vermis, lobus cranialis cerebelli and pedunculus cerebellaris medius); cerebellopontine angle with internal auditory canal; membranous labyrinth (cochlea, vestibulum and semicircular canals); and the brain stem.

**Table 1:** Rating scales for the three criteria: diagnostic usefulness, artifacts and image quality.

	Diagnostic usefulness	Artifacts	Image quality
**4**	best picture quality	no artifacts visible	best
**3**	diagnostic image quality (assessable)	artifacts present; no impaired assessment of the anatomic structures	good
**2**	inadequate	strong artifacts, limited evaluation of anatomic structures	insufficient
**1**	completely unusable	very distinctive artifacts, evaluation of anatomic structures not possible	completely deficient image quality

The presentation order of the MR images was in line with the mentioned acquisition: T1w MPR, axial T1w, axial T2w, coronal T2w, axial T1w with fat suppression, and CISS. The window values (picture contrast and brightness) could be adjusted by the examiner.

### Diagnostic usefulness

Diagnostic usefulness focused on the clinical detection, verification, and staging of possible brain pathology. The value was assessed using a 4-point ordinal rating scale: 4 = best picture quality; 3 = diagnostic image quality (assessable); 2 = inadequate; and 1 = completely unusable.

### Artifacts

Extent of artifacts was evaluated using a 4-point ordinal rating scale: 4 = no artifacts visible; 3 = artifacts present but do not impair assessment of the anatomic structures; 2 = strong artifacts with limited evaluation of the anatomic structures; 1 = very distinct artifacts that prevent evaluation of the anatomic structures.

### Image quality

Image quality was defined as how accurately the MR images could represent an intracranial pathology; this was based on the brightness and evenness of illumination, contrast, and resolution of the observed images. Evaluation of the image quality was based on the raters’ entire image impression, with special regard to the detection, verification, and staging of possible brain pathology. This also used a 4-point rating scale: 4 = best, 3 = good, 2 = insufficient, and 1 = completely deficient image quality.

### Statistics

In order to determine significant differences in the three rated criteria between the MR images acquired with or without presence of the magnet, we used the one-sided sign test for the statistical analysis due to the small number of cadaver heads. The inter-rater-agreement between the two blinded raters (FW and LL) was tested with the Cohen’s Kappa. A p-value < 0.05 was considered statistically significant.

## Results

**Fig 2:**
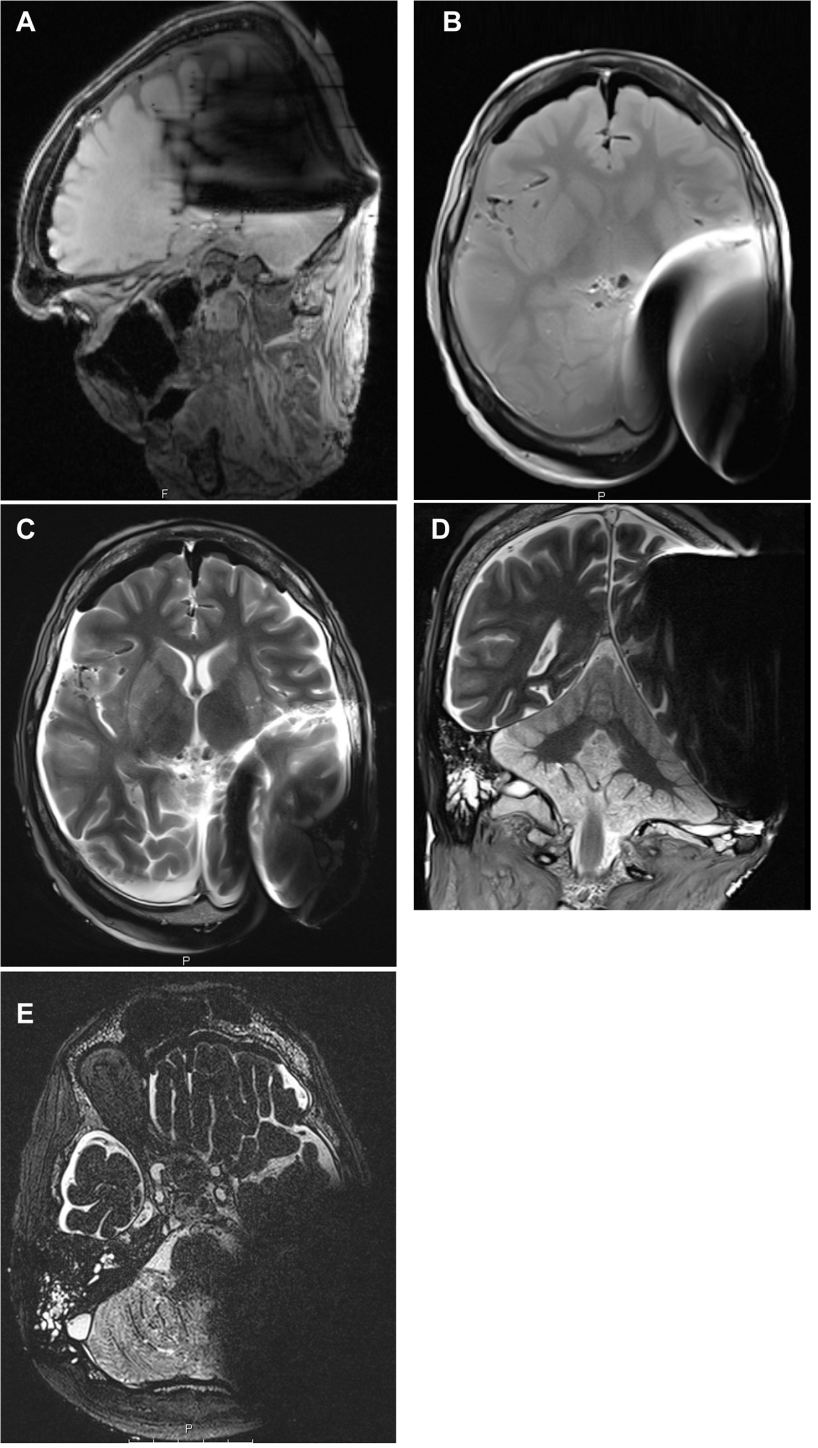
MR image series of one corpse head at 1.5T with magnet in situ. 2a: T1w MPR sagittal; 2b: T1w axial; 2c: T2w axial; 2d: T2w coronal;2e: CISS. The MR images show the maximum in diameter of the artefact in each sequence.

**Fig 3:**
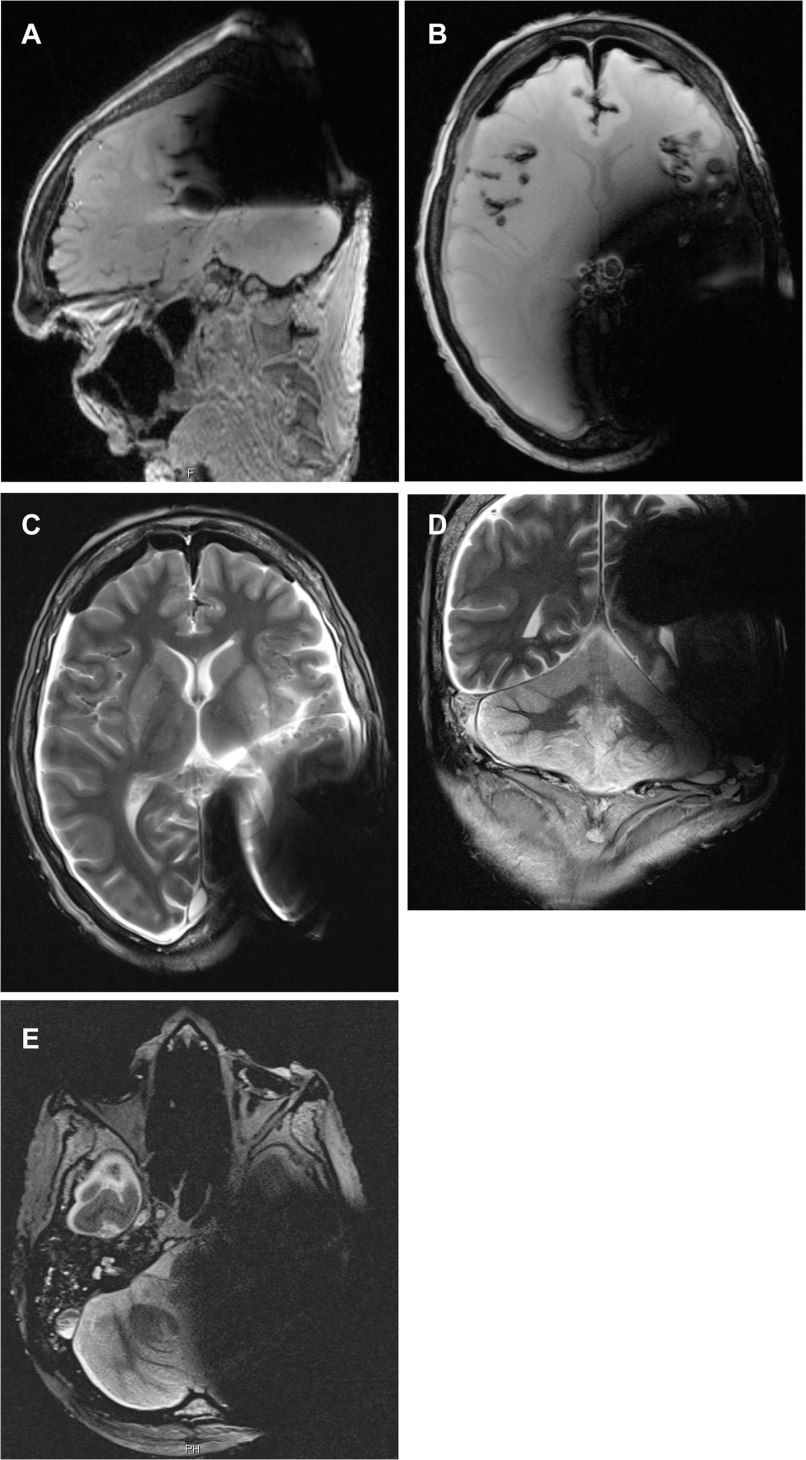
MR image series of one corpse head at 3T with magnet in situ. 3a: T1w MPR sagittal; 3b: T1w axial; 3c: T2w axial; 3d: T2w coronal; 3e: CISS. The MR images show the maximum in diameter of the artefact in each sequence.

For 1.5T and 3T, the MR images with the magnet in situ showed very distinctive artifacts consisting of a circular round signal void with an average maximal diameter of 5 cm (Figs 2 and 3) and a standard deviation (SD) of +/-1.5 cm, depending on the MR sequence. Particularly in the T1 MPR sequences and the CISS, pronounced shadowing artifacts were visible and covered the hemisphere and the cerebellum ipsilateral to the CI. Adjacent to the signal void, geometric distortions and reduced signal intensity were noted up to 8 cm (SD +/- 2cm) away from the center of the implant, which was also dependent on the MR sequence (T1 MPR and CISS). Overall, the acquired MR images with the magnet in situ were unusable for review of anatomical supra- and infratentorial brain structures or for clinical diagnosis on the implant side (inter-rater- reliability = 1, Table 2). On the contralateral (non-implanted) side the supra- and infratentorial brain structures were not layered by artifacts at 1.5T and 3T, and were considered to have best image quality and best picture quality for diagnostic usefulness (inter-rater-agreement = 1).

**Table 2:** Summary of the visibility of the rated anatomical structures at the side of the cochlea implant; with and without magnet at 1.5T and 3T.

Anatomical structure implanted side	1.5T MR magnet in situ	1.5T MR without magnet	3T MR magnet in situ	3T MR without magnet
temporal lobe	limited assessable	assessable	not assessable	assessable
parietal lobe	limited assessable	assessable	not assessable	limited assessable
occipital lobe	limited assessable	assessable	not assessable	limited assessable
vermis cerebellaris	not assessable	assessable	not assessable	assessable
lobus cranialis cerebelli	not assessable	assessable	not assessable	limited assessable
pedunculus cerebellaris medius	not assessable	assessable	not assessable	assessable
cerebellopontine angle	not assessable	assessable	not assessable	assessable
internal auditory canal	not assessable	assessable	not assessable	assessable
cochlea	not assessable	assessable	not assessable	assessable
vestibulum	not assessable	assessable	not assessable	assessable
semicircular canals	not assessable	assessable	not assessable	assessable
brain stem	limited assessable	assessable	not assessable	assessable

After removal of the magnet the quality of the obtained images at 1.5T and 3T improved significantly, with small (maximum diameter 1.0 cm on 1.5T and 1.5 cm on 3T (SD +/- 0.5 cm)) residual artifacts on the implanted side. These residual artifacts masked the peripheral lateral parietal and occipital lobes, the parietal and occipital bone, the parietal and occipital subcutaneous tissue and the lateral cranial lobus cranialis cerebellaris (Table 2). This situation was comparable to the MR scans with magnet in situ, which also showed stronger artifacts on the T1 MPR and CISS.

**Fig 4:**
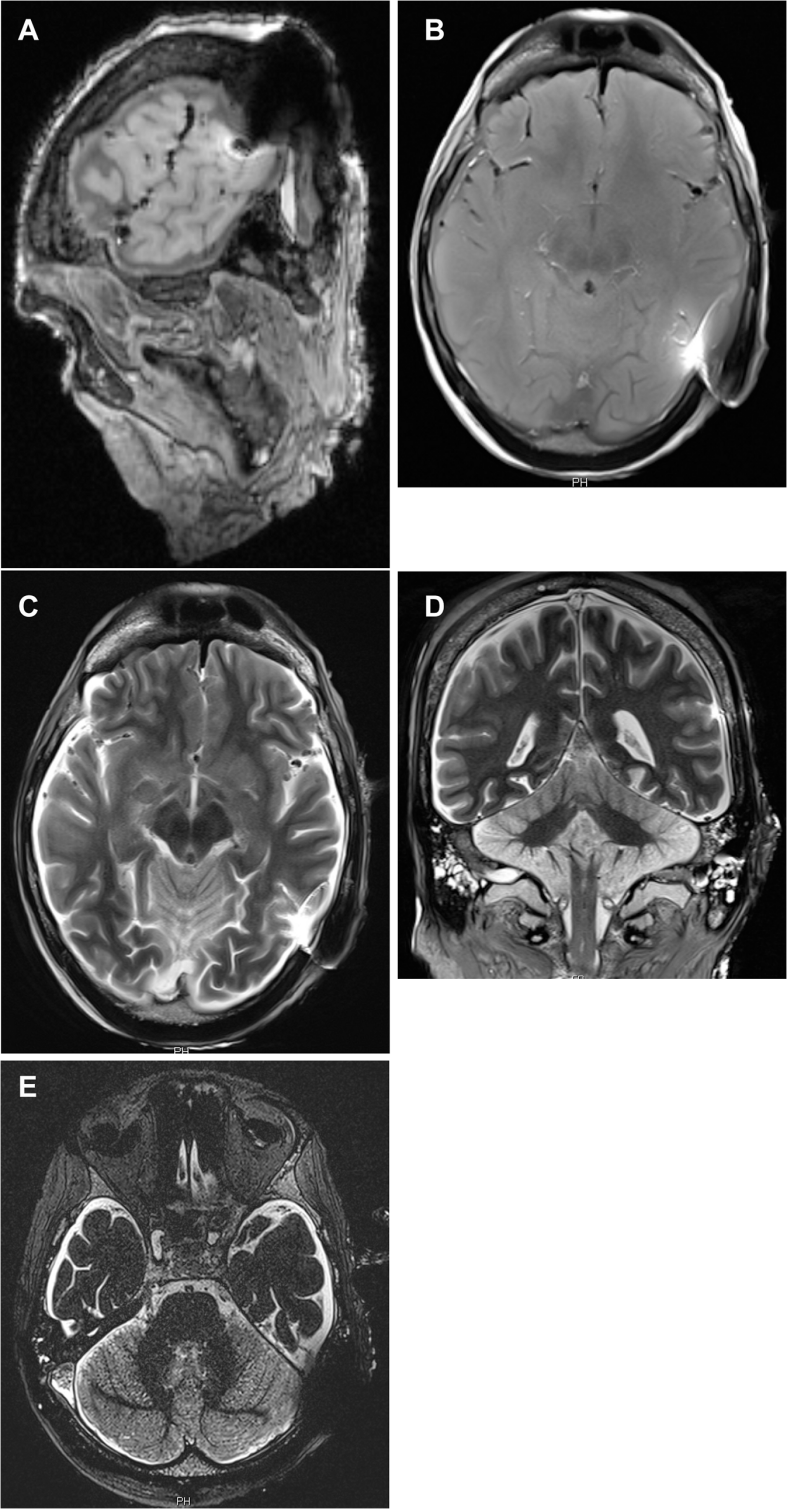
MR series of corpse head at 1.5T after magnet removal with non-magnetic spacer in situ. 4a: T1w MPR sagittal; 4b: T1w axial; 4c: T2w axial; 4d: T2w coronal; 4e: CISS.

**Fig 5:**
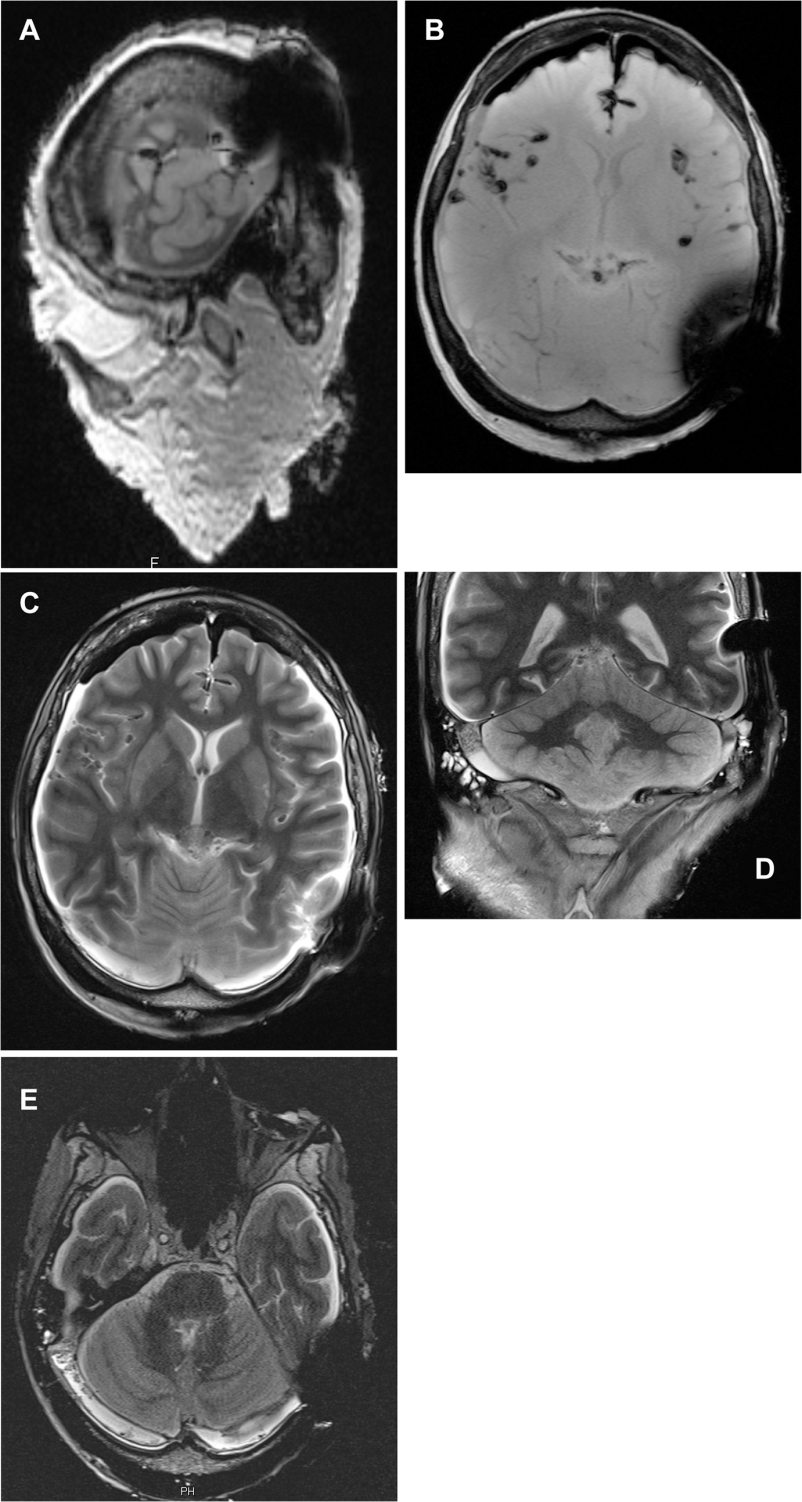
MR series of corpse head at 3T after magnet removal with non-magnetic spacer in situ. 5a: T1w MPR sagittal; 5b: T1w axial; 5c: T2w axial; 5d: T2w coronal; 5e: CISS. The MR images show the maximum diameter of the artefact in each sequence.

The occipital lobe, the vermis and pedunculus cerebellaris medius, the cerebellopontine angle with internal auditory canal, the membranous labyrinth (including cochlea, vestibulum and semicircular canals), and the brain stem were free of signal voids (Figs 4 and 5, Table 2).

In summary, the MR images after magnet removal at 1.5T and 3T enabled good image quality and diagnostic usefulness for the clinical practice and the evaluation of brain pathologies in CI carriers on the implanted side (Table 2). For the assessment of intracranial pathologies on the non-implanted side, a diagnostic-quality image is achievable at 1.5T and 3T even with the magnet in situ.

### Diagnostic usefulness

With the magnet in situ and a field strength of 1.5T, three of the five acquired image series of the corpse heads were inadequate (rated with 2) and the other two were completely unusable (rated with 1). After magnet removal all five image scans were diagnostic useful (rated with 3).

With the magnet in situ and a field strength of 3T, all images of the five cadavers were completely unusable (grade 1). After magnet removal both raters labeled the five image series with 3 (diagnostic image quality). The results are summarized in Table 3.

**Table 3:** Rating scale for the criteria diagnostic usefulness (inter-rater reliability: 1).

Score	1.5T MR with magnet	1.5T MRwithout magnet	3T MRwith magnet	3T MRwithout magnet
**4**	-	-	-	-
**3**	-	5	-	5
**2**	3	-	-	-
**1**	2	-	5	-

Improvement of the diagnostic usefulness after magnet removal at 1.5T and 3T was statistically significant (p-value 0.03), with no statistical difference between the different magnetic field strengths.

### Artifacts

All five of the assessed images at 1.5T and 3T with the CI magnet in situ had artifacts that affected the assessment of the anatomic structures. At 1.5T, 4 out of 5 images had strong artifacts with limited evaluation of the anatomic structures (grade 2); one image had very distinctive artifacts that prevented evaluation of the anatomic structures (grade 1). After removal of the magnet (i.e. exchange by the non-magnetic spacer) all images at 1.5T were graded 3 (artifacts present; no impaired assessment of the anatomic structures) by both reviewers.

At 3T all five MR scans with the magnet in situ had very distinctive artifacts that interfered with the evaluation of the anatomic structures (grade 1). After magnet removal the five MR image series were all scored 3 (artifacts present; no impaired assessment of the anatomic structures). The results are summarized in Table 4. The artifact reduction was statistically significant, with a p-value of 0.03 after the explantation of the magnet on 1.5T and 3T MR, with no difference between the two field strengths.

**Table 4:** Rating scale for the criteria artifacts (inter-rater reliability: 1).

Score	1.5T MR with magnet	1.5T MR without magnet	3T MRwith magnet	3T MRwithoutmagnet
**4**	-	-	-	-
**3**	-	5	-	5
**2**	4	-	-	-
**1**	1	-	5	-

### Image quality

With the magnet in situ and a field strength of 1.5T, three of the five cadavers showed insufficient image quality (grade 2) and two had completely deficient image quality (grade 1). After explantation of the magnet, the image series of all corpse heads were rated grade 3 (good image quality). The quality ratings for the 3T scans before the magnet removal were consistent for all five cadaver heads graded 1 (completely deficient image quality). At 3T without magnet the five acquired MR image series were rated as grade 3 (good image quality). The results are summarized in Table 5. A statistically significant difference was shown between the quality of the acquired images at 1.5T and 3T before and after the magnet removal (p-value 0.03). There was no statistical difference between the rating results after magnet removal at 1.5T and 3T.

**Table 5:** Rating scale for the criteria image quality (inter-rater reliability: 1).

Score	1.5T MR with magnet	1.5T MRwithout magnet	3T MRwith magnet	3T MRwithoutmagnet
**4**	-	-	-	-
**3**	-	5	-	5
**2**	3	-	-	-
**1**	2	-	5	-

The inter-rater-reliability was 1 for the three analyzed criteria–diagnostic usefulness, artifacts and image quality–after the magnet removal for the scans at 1.5T and 3T.

## Discussion

The MR images after magnet removal at 1.5T and 3T enabled the expected good image quality and diagnostic usefulness for the clinical practice and the evaluation of brain pathologies on the implanted side in CI carriers. The overall artifacts caused by the magnet in situ—more pronounced at 3T than at 1.5T —were significantly reduced after magnet removal at both field strengths. The acquired sequences at 1.5T and 3T after the magnet explantation indicated good visualization of the normal supra- and infratentorial brain structures without distortion. For assessment of intracranial pathologies on the non-implanted side, a diagnostic image quality is achievable even with the magnet in situ at 1.5T and 3T.

While aspects of CI magnet movements with potential CI damage or harm to the patients during or after MR scans are discussed elsewhere [23], the present human cadaver study analyzed diagnostic usefulness, artifacts and image quality of acquired MR brain series after cochlear implantation before and after magnet removal using a 1.5T and a 3T MR scanner. A previous report documented that CI devices with internal magnet removal are safe for 1.5T MR systems [24]. If the patient needs more than one MR examination the explanted magnet can be replaced with a titanium spacer and a new magnet can be placed when the patient has obtained all necessary MRIs [24,25,26].

This study confirmed previous results that image quality was significantly reduced on the implanted side by in situ magnet artifacts at both 1.5T and 3T, resulting in deficient image quality that was unusable for evaluating brain parenchyma, anatomy or underlying pathologies on the side of implant. Therefore, removal of the magnet for MR imaging of the brain, head, or neck region might be justified for initial or baseline imaging. In follow-up studies with known localization of the intracranial pathology (e.g. postoperative scans, benign brain tumors, low grade gliomas or demyelinating CNS disease) it might be not necessary to remove the magnet if the lesion is not in the previously documented area of cancellation artifact. This is an important fact especially for patients with neurofibromatosis type 2 who are offered cochlear implantation as standard treatment in the presence of an intact cochlear nerve and stable vestibular schwannomas [27]. Neurofibromatosis type 2 patients are required to have regular MR scanning to monitor disease progression, and, as a result, the safety and efficacy of repeated scanning can be improved by removal of the magnet.

Although magnet removal can be easily performed under local anesthesia via a small incision behind the ear, it may lead to additional costs and inconvenience for the patient. After completion of the MR scan the magnet can be repositioned manually with the magnetic insertion tool. The procedures to remove and to re-insert the magnet take about 15 minutes, from application of local anesthesia until the final sterile wound dressing. To avoid potential excessive scarring and adhesions that may occur after this minor procedure, the MED-EL Mi1200 SYNCHRONY cochlear implant includes a protective coating to prevent unwanted cellular adhesions. It is important to note that long-term study results concerning cellular adhesions in humans are not yet available. Furthermore, in vivo, infectious agents might spread along the incision into the cochlea, thus paving the way for intracranial infections such as meningitis. However, this potential complication should be avoidable in a sterile setting for magnet removal and re-implantation. Although we did not measure the temperature of the samples at the end of the MR examination, in our opinion the effect is inconsequential, as confirmed in prior studies [21].

Moreover, explantation of the magnet might result in weakening of the silicone pocket and thus increase the risk for a recurrence of dislocation [28,29]; however no long-term studies have been reported and the cited references refer to implant designs with removable magnets that are fundamentally different from the removable magnet design in the current study. The conical design of the MED-EL magnet is designed to avoid weakening of the silicone pocket.

Demagnetization of the magnet is described in the literature as a potential problem that depends on the position of the magnet in relation to the magnetic field of the scanner [23]. The magnet of the Synchrony implant is freely rotating and aligned parallel to the external magnetic field (Fig 1), thereby avoiding this problem.

Additionally, we are aware that some MR sequences are more exposed to cochlear implant magnet-induced field distortions and consequent more pronounced artifacts. The chosen MR examination protocol corresponds exactly to our institutional protocol for the assessment of pathologies in the cerebellopontine angle and temporal bone without contrast application. This closely simulates the methods used for neuroradiologic staging of patients with otovestibular complaints of unknown origin, sensorineural hearing loss, or for diagnostic and follow up of an intracochlear schwannoma; see, for example the case descriptions of Bittencourt at al. [30] and Tieleman et al. [31].

The chosen MR protocol is, in our opinion, consistent with standard ENT baseline imaging for temporal bone and internal auditory canal pathologies. This protocol is essential in the work-up of tumors and inflammatory diseases, such as the previously mentioned schwannomas, epidermoids, cholesterol granulomas, paragangliomas and transtegmental masses.

In accordance with the recently published work of Todt et al [32] we noticed that the artifacts are highly dependent on the technical scanning MR sequences used. In particular, the 3D CISS and the T1w MPR enlarge artifacts. But after magnet removal both sequences are suitable to evaluate pathologies of the cerebellopontine angle and to exclude, for example, the reoccurrence of a vestibular schwannoma within the inner ear or the internal auditory canal. An intralabyrinthine hemorrhage may also be solely depicted by unenhancend T1w MPR [33]. The 3D CISS is a standard part of a complete posterior fossa and internal auditory canal protocol, and is a mainstay for imaging of the cisternal cranial nerves at the cerebellopontine angle and internal auditory canal [34].

Todt et al. [32] also recommend a more horizontal and posterior position of the magnet for evaluation of the internal auditory canal and the labyrinth. In our opinion this is worth discussion because the authors focused exclusively on the ipsilateral infratentorial internal auditory canal and inner ear structures. We showed in our study that even with a standard retroauricular localization (nasion-ear angle) of the implant, an evaluation of the inner ear structures on the implanted side is possible after removal of the magnet. In consequence, the ipsilateral supratentorial brain structures must be more affected by artifacts due to the more horizontal and posterior implant position. Unfortunately, Todt et al. [32] do not discuss this issue. Additionally, the authors described the magnet dislocation at 3T as a yet unsolved problem [32]. Cochlear implants with a free rotating magnet aligning parallel to the external magnetic field solves this problem, and also solves the potential problem of magnet demagnetization.

In our opinion the removal of the magnet is a good method to reduce the artifacts, independent of the MR field strength, and to significantly avoid restrictions on the possible diagnosis of cerebral pathologies, including pathologies that are outside the area of the internal auditory canal and the inner ear structures of the implanted side. Besides the MR indication there is no other reason for a magnet removal; from the perspective of a CI carrier, magnet removal is disadvantageous in all conditions except MR scanning.

Our study is limited by the small sample size. We tested only the MED-EL Mi1200 SYNCHRONY cochlear implant system. Our results cannot be generalized to the other implant types such as Cochlear (Sydney, Australia), Advanced Bionics (Stäfa, Switzerland) and Neurelec’s device (Oticon Medical, Askim, Sweden).

Our small cadaver cohort showed, however, a good diagnostic value and good MR image quality with minimal artifacts on the implanted side at 1.5T and 3T after magnet removal.

## Conclusions

In conclusion, MR brain, head, and neck imaging can be performed in cochlear implant carriers at 1.5T and 3T without sacrificing diagnostic imaging quality, provided that the free rotating magnet is removed. On the implanted side, images of intracranial supra- and infratentorial brain pathologies are clinically more valuable after magnet removal as compared to diagnostic MR imaging with the magnet in situ. Removal of the magnet is advised for initial or baseline imaging. We recommend preserving the magnet in situ in patients with a known intracranial pathology outside of the artifact area.
